# Personalized Survival Prediction of Patients With Acute Myeloblastic Leukemia Using Gene Expression Profiling

**DOI:** 10.3389/fonc.2021.657191

**Published:** 2021-03-29

**Authors:** Adrián Mosquera Orgueira, Andrés Peleteiro Raíndo, Miguel Cid López, José Ángel Díaz Arias, Marta Sonia González Pérez, Beatriz Antelo Rodríguez, Natalia Alonso Vence, Laura Bao Pérez, Roi Ferreiro Ferro, Manuel Albors Ferreiro, Aitor Abuín Blanco, Emilia Fontanes Trabazo, Claudio Cerchione, Giovanni Martinnelli, Pau Montesinos Fernández, Manuel Mateo Pérez Encinas, José Luis Bello López

**Affiliations:** ^1^ University Hospital of Santiago de Compostela (SERGAS), Department of Hematology, Santiago de Compostela, Spain; ^2^ Health Research Institute of Santiago de Compostela, Grupo de Investigación en Síndromes Linfoproliferativos, Santiago de Compostela, Spain; ^3^ Departamento de Medicina, University of Santiago de Compostela, Santiago de Compostela, Spain; ^4^ Hematology Unit, Istituto Tumori della Romagna IRST IRCCS, Meldola, Italy; ^5^ Hospital Universitari i Politècnic La Fe, Department of Hematology, Valencia, Spain

**Keywords:** acute myeloid leukemia, cancer, survival, machine learning, gene expression, prognosis

## Abstract

Acute Myeloid Leukemia (AML) is a heterogeneous neoplasm characterized by cytogenetic and molecular alterations that drive patient prognosis. Currently established risk stratification guidelines show a moderate predictive accuracy, and newer tools that integrate multiple molecular variables have proven to provide better results. In this report, we aimed to create a new machine learning model of AML survival using gene expression data. We used gene expression data from two publicly available cohorts in order to create and validate a random forest predictor of survival, which we named ST-123. The most important variables in the model were age and the expression of *KDM5B* and *LAPTM4B*, two genes previously associated with the biology and prognostication of myeloid neoplasms. This classifier achieved high concordance indexes in the training and validation sets (0.7228 and 0.6988, respectively), and predictions were particularly accurate in patients at the highest risk of death. Additionally, ST-123 provided significant prognostic improvements in patients with high-risk mutations. Our results indicate that survival of patients with AML can be predicted to a great extent by applying machine learning tools to transcriptomic data, and that such predictions are particularly precise among patients with high-risk mutations.

## Introduction

Acute Myeloid Leukemia (AML) is a heterogeneous neoplasm characterized by cytogenetic and molecular alterations that drive patient prognosis. Currently established AML risk stratification guidelines, like the *European Leukemia Net* (ELN) risk classification (1), are based primarily on a limited number of cytogenetic and molecular variables. However, these guidelines don’t take into account the whole mutational profile of AML, the different layers of biological complexity in the tumor and the complex intertwining between patient outcomes and the complexity of molecular interactions. Therefore, there is substantial room for improving predictions of AML survival. In this report we present a new machine learning model of AML patient survival based on gene expression data, which achieves high predictability independently of high risk mutations.

In the last years, the emergence of artificial intelligence has brought new expectations to the field of medicine, particularly for disease diagnosis and prognostication. Machine learning (ML) is a field of artificial intelligence that performs outcome prediction based on complex interactions between multiple variables by making little assumptions about the relationship between the dependent and independent variables (2). In ML, a model is trained with examples and not programmed with human-made rules (3). The implementation of ML-based survival models is becoming increasingly popular in order to provide patient-centered risk information that can assist both the clinician and the patient.

Survival prediction of AML patients has been extensively improved in the last decades. Several biomarker panels based on next-generation sequencing of multiple recurrently mutated or aberrantly expressed genes have been proposed to facilitate improved prognostic stratification. Although multiple somatic alterations have been associated with patient outcome, such as those in *NPM1*, *CEBPA*, *FLT3*, *IDH1*, *IDH2*, *KIT*, *WT1* and *RUNX1* (4), only mutations a few mutations are broadly employed in current clinical routine (5). Multiple studies have proposed novel biomarker panels and personalized survival prediction models aimed to improve AML prognostication. Sherve et al. (6) developed a novel prognostic model that incorporates clinical, cytogenetic and mutational data to determine personalized outcomes for each particular patient (6). In the same line, Patkar et al. (7) created a scoring model that provides a mechanism to risk stratify AML patients with mutated *NPM1* (7); whereas Gerstung et al. (8) reported statistical models that can generate personally tailored clinical decision support from all of the available prognostic information arising from a knowledge bank of AML cases (8). These studies evidence that the application of ML to clinical and molecular data has the potential to predict patient outcomes since the moment of diagnosis, and therefore it may help to improve therapeutic strategies in the field of AML.

In the present study, we applied ML algorithms to gene expression data from AML cases in order to create new individualized models of survival based on retrospective data, and to understand their relationship with high-risk mutations.

## Methods

Two databases available in the *Gene Expression Omnibus* were used for model training and validation. The GSE37642 database was used for training, as it contains gene expression data from 562 adult patients diagnosed with AML who were treated in the multicenter phase III AMLCG-1999 trial. Median age was 45 years (range 18-85 years, 32% aged ≥ 65). The GSE68833 database was used for validation, and it contains gene expression data from 137 adult patients with AML included in *The Cancer Genome Atlas* (TCGA) cohort, with a median age of 59 years (range 18-88 years, 33% aged ≥ 65). Both databases used partially overlapping microarray chips, so that we rank-normalized the log2-transformed expression estimates and selected a set of 44,366 common gene expression probes between both cohorts.

### Unsupervised Gene Expression Clusterization

Briefly, the *Mclust* algorithm (9) was used in order to detect the 2 most likely clusters of patients according to the expression of each probe (*Mclust* function, parameter G = 2). Briefly, the *Mclust* algorithm determines the most likely set of clusters according to geometric properties (distribution, volume, and shape). An expectation-maximization algorithm is used for maximum likelihood estimation, and the best model is selected according to Bayes information criteria. The association of each of these probe-level clusters with overall survival was calculated using cox regression. Thereafter, those probes whose clusterization was significantly associated with survival (Bonferroni adjusted *p-*value < 0.05) were selected for multivariate clusterization using the same *Mclust* algorithm. Cluster prediction was performed on the test set using parameters estimated in the training cohort, and cox regression was used to verify the association of this clusterization with overall survival. The Shoenfeld’s test was used to assess the proportional hazards assumption.

### Survival Analysis

We analyzed gene expression association with overall survival using cox regression implemented in R. Assumption of proportional hazards was checked with Schoenfeld’s method.

### Random Forest Survival Analysis

We started our analysis by testing the association of each probe with overall survival in the training set using multivariate cox regression. Schoenfeld’s method was used to assess the proportional hazards assumption.

Random forest survival models were created with the *rfsrc* function implemented in the *randomForestSRC* package in R (10). We decided to use this type of model because, in contrast with deep networks, random forest can quantify the relative importance of each variable, and thus enable the filtering of low-importance variables for model reduction and performance improvement. Parameter tuning was performed using the *tune.rfscr* function, which optimizes the *mtry* and nnodes variables. Random forests were implemented on survival data of the training cohort. Bootstrapping without replacement was performed with the default *by.node* protocol. Continuous rank probability score (CRPS) was calculated as the integrated Brier score divided by time, and represents the average squared distances between the observed survival status and the predicted survival probability at each time point. CRPS is always a number between 0 and 1, being 0 the best possible result. Survival prediction on the test cohort was performed using the *predict.rfsrc* function with default parameters. Harrel’s concordance index (c-index) was used to assess model discriminative power on the bootstrapped training set and on the test set. C-index reflects to what extent a model predicts the order of events (e.g., deaths) in a cohort. C-indexes below 0.5 indicate poor prediction accuracy, c-indexes near 0.5 indicate random guessing and c-indexes of 1 represent perfect prediction.

Variable selection was selected by fitting age-adjusted cox regression models of overall survival in the training set. We initially analyzed different sets of genes in order to select the best input sets. Afterwards, we selected different sets of genes according to their multiple testing adjusted p-values; either False Discovery Rate (FDR) or Bonferroni-adjusted p-values below 0.01, 0.05, 0.1. Variable reduction was performed by iteratively removing those variables with low importance. Variable importance was calculated with the *vimp* function, and we iteratively removed those samples with negative or low weight (importance < 1 × 10^−4^). We replicated the models in the independent set.

## Results

### Model Selection

**Figure 1:**
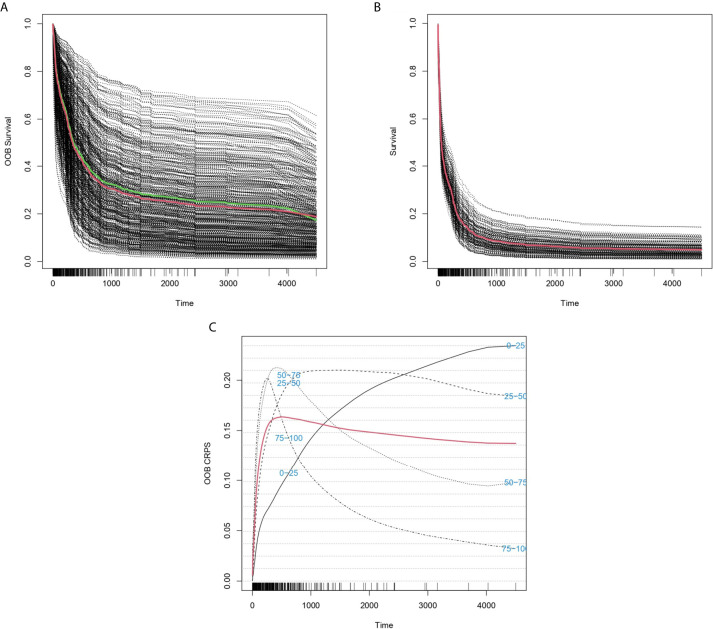
Predicted individual survival curves according to the best random forests model. **(A)** Out-of-bag survival curves predicted for patients within the training cohort. The thick red line represents overall ensemble survival and the thick green line indicates the Nelson-Aalen estimator. **(B)** Individual survival curves predicted for patients within the test cohort. The thick red line represents overall ensemble survival. **(C)** Representation of out-of-bag CRPS over time. Red line is the overall CRPS. Additionally, stratified CRPS by quarters of predicted ensemble mortality are provided. Vertical lines above the x axis represent death events.

We created six different survival models by taking into consideration different sets of transcripts at various thresholds of statistical significance. Importantly, all models achieved c-indexes > 0.67 in both the training and the validation sets. The best model contained 123 variables (absolute age, age ≥ 65, and the expression of 121 probes) (**Supplementary Table 1**). We name this model *Stellae-123* (ST-123). ST-123 achieved c-indexes of 0.7228 in the training set and 0.6988 in the test set, indicating a high reproducibility of the personalized risk prediction (**Figures 1A, B**). Apart from age, the most important variables in ST-123 were the expression of *KDM5B* and *LAPTM4B* (**Supplementary Table 2**).

### Performance of ST-123 Over Time

**Figure 2:**
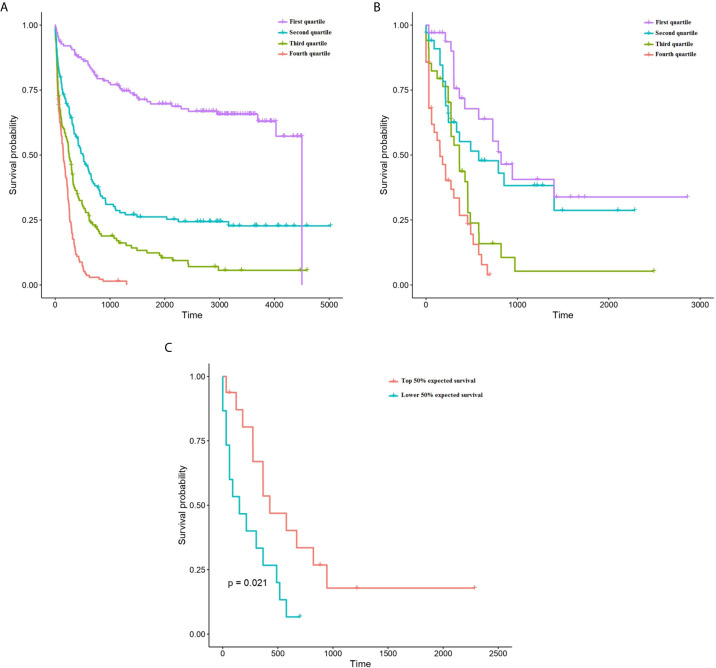
Kaplan-Meyer plots representing the survival of patients depending on their classification to each quartile of predicted survival by ST-123 in the training **(A)** and validation **(B)** cohorts. **(C)** Kaplan-Meyer plots representing the outcomes of patients affected by high-risk mutations (*TP53* mutation/deletion, *ASXL1* mutation or *RUNX1* mutation) depending on their classification to the upper or lower median of predicted mortality by ST-123 in the validation set.

The predictive accuracy of ST-123 was sustained over time, as reflected by the similar c-indexes obtained at 5 years of follow-up. Furthermore, mortality predictions were more accurate among those patients at high-risk of death according to the model. Additionally, CRPS plots indicate a rapid loss of predictability in the first days after diagnosis followed by an stabilization of survival predictions over time (**Figure 1C**). Indeed, c-indexes were less predictive for patients who died in the first days since diagnosis (**Table 1**). (ST-123 could clearly stratify patients in 4 different quartiles with different and reproducible mortality rates (**Figures 2A, B**).

**Table 1:** C-indexes of ST-123 after restricting the analysis to different time points since diagnosis.

Days since diagnosis	C-index (Training set)	C-index (Test set)
**<10**	68.65	59.66
**<20**	70.13	59.54
**<30**	71.88	60.19
**<60**	73.86	62.13
**<100**	74.06	63.11
**>100**	71.94	69.17

### ST-123 Performance in Patients With High-Risk Mutations

We tested the performance of the classifier in patients with high risk mutations in the TCGA cohort (validation set). We selected cases harboring *TP53* mutation and/or deletion (16 patients), *RUNX1* mutation (14 patients) and *ASXL1* mutation (6 patients) (1). We didn’t include FLT3-ITD cases because this mutation type was not reported in the TCGA cohort. Using two-year survival predictions, we could observe a statistically significant association with overall survival within these high-risk patients (p-value 4.04 x 10^-4^, age-adjusted p-value 2.16x 10^-3^) (**Figure 2C**). Indeed, both cox models achieved high concordance measures: c-indexes of 0.774 (standard error 0.052) and 0.767 (standard error 0.042), respectively.

## Discussion

The concordance measure of ST-123 is substantially superior to that described for the ELN risk classification (0.59) (6), which suggests an increased performance. Furthermore, ST-123 seems particularly useful to better stratify high-risk patients. These results are in line with other ML-based models of survival based on mutational data described so far (8), which emphasizes the possibility of improving risk stratification in AML by implementing artificial intelligence. Although other prognostic models, such as that published by *Sherve et al.* (6), report a higher predictive accuracy, these also incorporate several baseline clinical variables. Indeed, CRPS plots indicate that the highest loss of prediction accuracy in our model occurs early after disease diagnosis, which reflects the fact that clinical variables are the main predictors of early mortality in AML (8). On the contrary, we can see how the CRPS curve of our model rapidly reaches a plateau, maintaining values ∿0.15 for long periods of time, reflecting the stability of the predictions over time. It seems reasonable that the incorporation of clinical variables (such as performance status and leukocytosis) will improve the overall predictive accuracy of ST-123 by identifying those patients at risk of early death. On the contrary, little contribution of mutations to gene expression patterns has been observed in myelodysplastic syndromes, and this issue needs to be addressed in AML (11).

It has been hypothesized that a better risk stratification based on the integration of different layers of clinical and biological complexity could, for example, reduce the number of allogeneic stem cell transplants performed in patients with AML by 20-25% while maintaining the same overall survival rate as that with the current treatment recommendations (8). In the same line, high-risk older patients might benefit from innovative drugs and drug combinations (12). This could help to restrict the most aggressive or innovative treatments to those AML patients who really are at the highest risk of death. Therefore, it becomes increasingly important to incorporate these new personalized models of survival in the risk stratification of AML, rather than keep relying on imperfect predefined risk groups. There is a need to analyze all these predictors in systematic and independent cohorts in order to provide further evidence about their effectiveness, and also to potentially integrate their individual predictive accuracies in more precise estimations of patient outcomes (13). Importantly, accumulated experience indicates that dichotomized molecular classifiers in AML can be reproduced in an unbiased and independent approach (14), but further efforts need to be made to compare the performance of personalized predictors such as ours.

From a biological point of view, the most important genes in ST-123 were *KDM5B* and *LAPTM4B*. Not surprisingly, both genes are deeply vinculated with carcinogenesis. *KDM5B* (Lysine Demethylase 5B) encodes a master epigenetic regulator of H3K4 methylation that regulates the expression of several oncogenes and tumor suppressors during carcinogenesis (15). *KDM5B* downregulates the oncogenic potential of leukemic stem cells by inducing H3K4-specific demethylation in murine and human MLL-rearranged AML cells, thereby promoting cell differentiation (16). Indeed, the KDM5B protein is the target of various inhibitors that are under study for the treatment of cancer (17). In the same line, the oncogene *LAPTM4B* (Lysosomal Protein Transmembrane 4 Beta) plays several roles in carcinogenesis, such as promoting tumor growth and metastasis, inhibiting apoptosis, initiating autophagy and driving multidrug resistance mechanisms (18). Interestingly, the expression of *LTPM4B* has been shown to be prognostic in myelodysplastic syndromes and in different types of solid tumors (19, 20).

In conclusion, our results indicate that survival of patients with AML can be predicted by applying ML tools to transcriptomic data, and that such predictions are particularly precise among patients with high-risk mutations. The possibility to enrich transcriptomic models such as ours with clinical and mutational data will lead to a more precise and holistic prediction of AML survival, paving the way for the development of new personalized treatment strategies.

## Data Availability Statement

The datasets presented in this study can be found in online repositories. The names of the repository/repositories and accession number(s) can be found in the article/**Supplementary Material**.

## Author Contributions

AM had the idea and performed the research. AM, AP, MC, JD, MG, and BA analyzed the data and wrote the paper. NA, LB, RF, AA, MA, and EF analyzed the manuscript and made suggestions. CC, GM, PM, MM, and JL gave final consent for publication. All authors contributed to the article and approved the submitted version.

## Conflict of Interest

The authors declare that the research was conducted in the absence of any commercial or financial relationships that could be construed as a potential conflict of interest.

## Supplementary Material

Supplementary Table 1 Characteristics of the different random forest models created at the different significance thresholds. Error rates (1-concordance) are provided for predictions of survival at 1, 2, 3, 4, 5 and full follow-up periods.

Supplementary Table 2 Variables included in the final model along with their relative importance. For gene expression probes, we also report the type of input used (either continuous gene expression counts or *Mclust*-based binary clusterization).
